# Explainable artificial intelligence models using SHAP enhanced CatBoost, Bi-GRU with attention, and Tab Transformer

**DOI:** 10.1038/s41598-026-49961-7

**Published:** 2026-05-02

**Authors:** Markapurapu John Dana Ebinezer, Bondalapu Chaitanya Krishna

**Affiliations:** Department of CSE, Koneru Lakshmaiah Education Foundation, Vaddeswaram, India

**Keywords:** Insurance fraud detection, SHAP explainability, CatBoost, TabTransformation, Bi-GRU with attention, Engineering, Mathematics and computing

## Abstract

Insurance fraud detection remains challenging to predict in reality because claims data is often uneven among classes, and the information concerning claims is often multidimensional and nonhomogeneous. The present research used a unified evaluation framework to assess the predictive and interpretive capabilities of three distinct model families: CatBoost (tree-based ensemble learning), Bi-GRU with Attention (sequence-oriented learning), and TabTransformer (categorical feature contextual). The model families were tested using a standardised experimental protocol.The study is novel in the sense of a cross-model interpretability framework that unites Shapley Additive Explanation (SHAP)-based feature attribution with attention-based contextual analysis to enable a clear comparison of model reasoning between the suggested frameworks. The data on which the experiments were done consisted of 4,000 life insurance claims that were characterized in terms of 83 attributes. Common preprocessing procedures like missing values, scaling numerical variables, and selecting highly correlated variables were used before training the models. Experimentally, CatBoost is proven to be the most precise on legitimate claims, Bi-GRU is the most recall on fraudulent claims, and TabTransformer is the best in terms of tradeoff between accuracy, interpretability, and computational efficiency. Practical characteristics such as the quantity of claim, tenure in a policy, and diagnosis were repeatedly emphasized in both SHAP and attention analyses. Combined, the current research study provides a consistent and explainable benchmark that may be applied to conduct fraud detection research reliably and assist practitioners in choosing models that are accurate and understandable.

**Supplementary Information:**

The online version contains supplementary material available at 10.1038/s41598-026-49961-7.

## Introduction

Insurance fraud has become a major issue for both financial and healthcare systems. According to the findings of various international evaluations, fraud in businesses costs billions of dollars annually. It is estimated that insurance fraud is also eating almost 10% of overall healthcare spending^1,2^. As digital and automation have been growing, new gaps have emerged, which are exploited by fraudsters that force more financial burden on both the providers and the insurers. Past methods - primarily manual audits and rule-based fraud detection systems, although effective in earlier times, are not enough to meet the current complexity of fraud schemes^3,4^. These constraints show the necessity to have stronger, data-driven detection policies that can react to the increasing complexity of fraud. The recent advancement in machine learning (ML) and deep learning (DL) has substantially improved the fraud detection systems. Ensemble-based algorithms that show resilience in unbalanced classification issues, including Random Forest and XGBoost, are still widely used to handle claim data. In parallel, recurrent neural networks (RNNs) and their variant, such as gated recurrent units (GRU) and long short-term memory (LSTM), have shown value in capturing the sequence of claim histories, improving the detection of temporal fraud patterns. More recent work on transformer-based designs, such as TabTransformer, and SHAP with SHAP added to learn contextual embedding using categorical and numerical features, has shown encouraging results in tabular data applications. In view of the current industry demands, limitations in the literature^5–7^. The central research problem is as follows: Existing insurance fraud detection systems do not provide a unified, explainable AI framework that can systematically compare different categories of models, such as heterogeneous model families (ensemble, sequential, transformer), while ensuring transparent decision-making through model-agnostic interpretability tools such as SHAP. In particular, there is a limited use of model-agnostic tools like SHAP to ensure transparent decision-making across these models.

Explainable AI is increasingly important in the insurance sector, where analytical decisions directly affect claim settlement, provider evaluation, fraud investigation, and compliance with regulatory requirements. Transparent modelling not only strengthens stakeholders but also helps insurers to recognize practical fraud indicators, including unusual claim amounts, inconsistent diagnosis procedure pairs, and irregular provider level patterns. An environmental financial fraud necessitates studies in regulatory audits, and wider adoption of automated decision systems, an explainability-driven, multi-modal benchmarking, and studies both relevant and necessary^8^. The single evaluation of ensemble, sequential, and transformer-based models, thus, facilitates current research.

To overcome the above shortcomings, the following objectives will be the goals of this study:

1. To curate and preprocess a life insurance dataset (4000 claims) and (83 features) using reduced imputation, normalization, filtering features with correlation-based, and augmentation techniques to decrease imbalance and redundancy.
2. To Design and implement three model types of complementary fraud detectors, including CatBoost with SHAP, Bi-GRU with attention, and TabTransformer with SHAP, which are an ensemble, sequential, and transformer paradigm, respectively.
3. To perform a serious assessment with the help of confusion matrices, statistical performance measures, ROC-AUC, precision-recall curves, and suitable significant tests, make sure that there is both strength and balance in the comparison.
4. To add SHAP explainability to CatBoost and TabTransformer models, which augments clear feature attribution and serves to determine the influential factors that relate to fraud, including the size of claims, duration of the policy, and provider conduct.
5. To identify more effective and interpretable frameworks by use of multi-criteria analysis that allow the balancing of predictability, strength, explainability, clarity, and practical applicability in a real-life implementation in insurance applications.

### Related works

The recent research has focused primarily on how to enhance classification accuracy, whereas explainability, which is a critical element in both stakeholder trust and regulatory oversight, has been relatively rare. Further, comparative assessments of traditional, sequential, and transformer-based models in a single explainability-focused framework are also evidently missing, and represent a gap in the literature^9,10^.

In order to highlight the novelty of the current research, Table 1 outlines a benchmarking review of the recent literature on insurance and healthcare fraud detection. The comparison reveals that prior studies either utilized ensemble ML models or deep neural networks; not many have utilized model explainability tools such as SHAP, or provided a multi-model comparison analysis^11,12^.

A condensed benchmark of the recent literature on insurance and healthcare fraud detection is provided in Table 1 and shows the methodological variability and weakness of the previous literature. The initial methods, including rule-based analytics and traditional data mining methods^13^, offered some basic understanding, but failed to scale and were unable to detect complicated fraud patterns. Predictive accuracy of ensemble learning models such as XGBoost and CatBoost was better on tabular data, but neither of these studies used fraud-specific interpretability, and the decision paths of these models remain obscure to researchers^14,15^.

The classical machine learning pipelines based on SVM, Random Forest, and neural networks alleviated the problem of imbalance more or less, yet they still did not provide the sequential modelling functionality and the transparent attribution process. Fraud detection schemes based on deep learning showed better results in heterogeneous medical claims, but were black box and could not be explained.

Transformer-based architectures like Tab Transformer^16^ demonstrated potential in the modelling of categorical interactions, yet were not used on insurance fraud, nor did they have SHAP-based interpretations. More recent studies of interpretable deep learning^17^ focused on explainability but made no comparisons of heterogeneous model families within a single framework. In general, benchmarking in Table 1 suggests that all current research is based on predictive accuracy or single model classes, yet there is a dearth of research on the issues of transparent reasoning, cross-model evaluation, or SHAP-driven interpretability. This puts the gap in research of the current study in a clear way - a single, explainable benchmarking model that incorporates ensemble, sequential, and transformer models to detect fraud.

**Table 1:** Literature benchmarking an insurance/healthcare fraud detection.

References	Methodology	Dataset type	Key limitation	Contribution gap	Contribution / our contribution
^18^	Data mining and rule-based	Health care claims	Limited Scalability; no deep learning	Lack of advanced ML models	Introduce ML + DL comparative pipeline
^19^	XGBoost	Tabular	No Interpretability	Explainability missing	Extend with CatBoost + SHAP
^20^	CatBoost	Tabular	No fraud focus	Not benchmarked in insurance	Apply CatBoost + SHAP for fraud
^21^	SVM, RF, NN	Insurance claims	Poor Imbalance handling	No sequence modelling	Benchmark Bi-GRU + Attention
	Deep Learning	Healthcare fraud	Black-box	No transparency	SHAP-based interpretability
^22^	TabTransformer	Tabular categorical	General tabular task	No fraud application	Adapt TabTransformer + SHAP
^23^	Interpretable DL	Insurance claims	Limited benchmarking	No cross-model study	Multi-model comparative framework
Current study	CattBoost with SHAP Bi-GRU with Attention TabTransformer with SHAP	Life insurance claims (4000, 85 features)	-	-	Unified multi-model pipeline SHAP-based explainability statistical validation

The existing literature assesses the algorithms of fraud detection primarily based on the predicted accuracy, but the current study proposes a benchmarking method that focuses on explainability in an experimental study. The study brings together three decent models with attention – CatBoost with SHAP (ensemble learning), Bi-GRU with attention (sequential learning), and TabTransformer with SHAP (Contextual embedding). This work has contributed to more than just a comparison of models; SHAP-explanations can be directly compared across different models by applying the established qualification of cross-model interpretability, where the evaluation protocol is the same. The other contribution is the presentation of a dual-layer interpretability mechanism, in this case, feature-level SHAP attribution and sequence-level attention visualizations, and the ensuing findings are evaluated by Wilcoxon signed-rank significance testing. The combination of transparency, model diversity, and statistical validation makes the study take a step further in engineering solutions and offers a more generalizable methodological framework of explainable insurance fraud detection - a field previously covered in the literature.

Table 1 and the literature review indicate that there are several shortcomings:

Previous literature is more focused on predictive performance and minimal consideration of explainability, which makes the process of decision-making obscure.

Existing works offer a focus on single model families (ensemble ML or DL) without comparative benchmarks across paradigms.

SHAP explainability has been fully investigated in the context of integrating it into various models (tree-based and transformer-based) to detect insurance fraud.

Very little research has tried to combine traditional, intermediate, and advanced architectures into a single experimental setup.

The existing techniques cannot strike a balance between predictive accuracy, interpretability, and generalizability when heterogeneous insurance data are used. As a result, integrated, elucidated, and comparative evaluation models that integrate tree-based, sequential, transformer-based models have not yet been achieved, in order to face reliable fraud detection. The limitation of the implementation of the real situation in the insurance industry, where transparency and accountability should be accessible, can also be attributed to the fact that SHAP was not popular in the diverse models^24,25^.

## Methodology

### Dataset preparation and preprocessing

The data of this study was developed in-house to replicate the activity of insurance claims in the real world, as well as the characteristics of fraud. It comprises *N* = 4000 records, with each record being represented as a distinctive feature vector. Every record is denoted as a feature vector. x^((i))∈R^d where d = 83 indicates the raw features and i = 1, 2. N. The desired output is a binary class label y^ ((i)) = 0,1 such that y (i) = 1 corresponds to a fraudulent claim and y (i) = 0 corresponds to a legitimate claim. The total percentage of fraud is approximately 18%, and this corresponds to the widely reported fraud rate in insurance literature (Eqs. (1) & (2)).

To maintain the structure closer to the manner in which insurers store information, each attribute was put into a logical group, which represents a functional element or actual claim data. The 83 features were categorized into six broad groups as indicated in Table 2: demographics, financial attributes, procedural-related details, diagnostic codes, provider behaviour, and other temporal aspects. All attributes are listed, with the data types, category assignment, and a concise semantic description, in supplementary file 1.

**Table 2:** Data set feature categories.

Category	Description	Example feature
Demographics	Patient-related info	Age, gender, and Region
Financial	Monetary claim characteristics	Claim amount, procedure cost, reimbursement
Procedural	Medical Procedure rated defaults	Diagnosis code, Diagnosis group
Provider	Behavioural and statistical provider info	Provider ID, Claim Frequency, Speciality
Temporal	Time-dependent attributes	Claim date, length of stay, Treatment period

1 $$\:D=\left\{\left({x}^{\left(i\right)},\:{y}^{\left(i\right)}\right)\:\:\:\:\:\:\left|i=\mathrm{1,2},3,\dots\:.N\right|\right\}$$

2 $$\:{x}^{i}=\left[{x}_{1}^{\left(i\right)},\:{x}_{2}^{\left(i\right)}\dots\:\:{x}_{83}^{\left(i\right)}\right]$$

### Data simulation framework and statistical design

To recapitulate the life insurance claim behavior in the real world and maintain the control of the experimental environment simultaneously, a synthetic data set was developed that supplied *N* = 4000 records of claims, where about 18% were deemed fake. Each record contained 83 features that were grouped into six major domains: demographic details, financial variables, procedural information, diagnostic or procedural, provider, Information behavior, and temporal. The data set was generated based on a replicable stochastic model to capture the statistical relationships observed in real portfolios of insurance claims that were reasonably modeled. Deterministic reproducibility was set to 42 to seed the randomness. Assignments of feature-level distributions were made using domain-driven priors using public insurance studies. Marginal distributions were defined as follows:

- The normal and categorical distribution of demographic variables (age, region, gender) was sampled according to the census proportions.
- A log-normal distribution ( m = 10, s = 0.4) was used to create financial features (claim amount, reimbursement, procedure cost) with a right-tailed variability.
- The procedural and diagnostic attributes were represented as multimodal variables with weight proportions of the high-cost and low-cost services.
- Poisson (λ = 3) and categorical distributions were used to draw provider-level statistics (claim frequency, specialization).
- Temporal variables (length of stay, claim duration) were gamma-distributed ( 2, 15 ) that reflected the skewed duration of hospitalization.

The covariance of features was presented in an organized covariance matrix that had a mean Pearson correlation coefficient (τ = 0.35) among the financially related variables (e.g., claim amount and reimbursement ratio). Continuous variables were perturbed with additive Gaussian noise 0.052) to mimic natural variability in real-claim data. The rule-based hybrid scoring scheme was used to assign fraud labels, and looked at combinations of features including unusually claim-to-premium ratio (> 1.8), repeated provider identifiers, and unusual diagnosis procedure pairs. The records that had values that were above a composite anomaly threshold were tagged as fraudulent (y = 1). This method was used to ensure a fraudulent distribution was always statistically close to that which is generally reported in an industry context.

**Algorithm 1:**
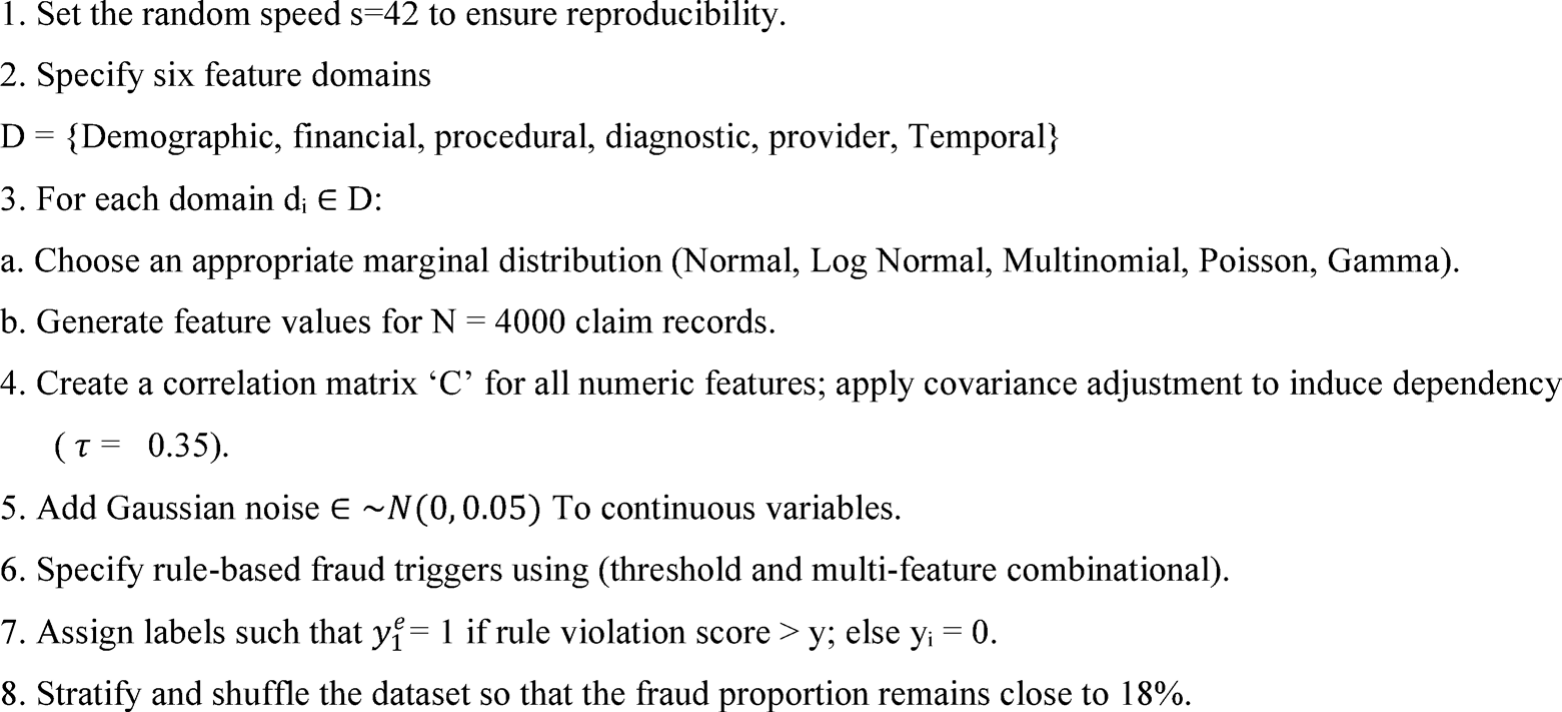
Synthetic data generation framework.

### Dataset scale and validation justification

The dataset constructed was compared to known benchmark datasets, including CMS Medicare provider utilization and payment details, and the Kaggle health insurance cross-sell dataset to evaluate its realism and scope. Such open datasets tend to have fewer attributes and can only be transactional or policy-based. However, the dataset constructed in this research includes 83 heterogeneous characteristics that include demographic, procedural, financial, provider-specific, and claim-related characteristics. This grandiose multidimensional framework permits a more vivid description of cross-feature relationships and diverse patterns that are usually related to fraud behavior.

The process of generating the dataset had a number of methodological challenges. To begin with, realistic claim behavior when strict privacy demands were imposed implied the production of distributions that would mimic the behavior of real insurance without replicating actual records. Second, have difficulty in joint correlations between mixed-type variables, i.e., between the interactions between age and the policy or dependency between a provider and the cost of claiming, which had to be validated and preserved through many repeated multivariate correlations. A reasonable ratio of fraud and non-fraud (say 18:82) also necessitated the trade-off between the non-frauds that are difficult to find in the real world and the required number of minority representatives to achieve convergence in the model. Also, though, the task of managing categorical and continuous variables in a single encoding and normalization configuration requires some finesse; otherwise, a feature will come to dominate the learning process.

To overcome the risk of overfitting that is likely to happen due to the usage of the small dataset, multiple methodological controls were employed:

(i) Five-fold cross-validation to keep the structure of imbalance between classes in each fold.
(ii) Deep learning, early stopping, and dropout regularization.
(iii) Complexity normalized comparison to make fair comparisons across the models.
(iv) External verification through public CMS Medicare provider utilization data to determine robustness in general.

All of these steps contribute to the statistical reliability despite using a small amount of data. The constant pattern of folds proves that the models had the real, authentic patterns of fraud, not the artifacts of data scale of artificial data creation.

Therefore, the suggested dataset is deemed to be sufficient to benchmark explainable deep learning architectures in controlled experimental conditions.

### Data cleaning and missing value imputation

Before modeling, missing values were treated to ensure dataset consistency. For each numerical feature $\:{x}_{K}\in\:R,\:$Missing values were imputed using the median of the respective column–wise median, as defined in Eq. (3):

3 $$\:{x}_{K}^{\left(i\right)}=\left\{\begin{array}{c}{x}_{K}^{\left(i\right)},\:\:\:\:\:\:\:\:\:if\:{x}_{K}^{\left(i\right)}\ne\:Null\:\:\:\:\:\:\:\\\:median\:\left({x}_{K}\right),\:otherwise\:\:\:\:\:\:\:\:\end{array}\right.$$

Categorical features $\:{x}_{j}\in\:{Z}^{t}$ The missing values were filled with a fixed token marked “unknown” to preserve the semantic structure of the data. This method avoids disturbing value distributions, ensuring that each instance retains the same feature dimensionality.

### Categorical encoding strategies

The dataset contains 35 categorical features. Based on model requirements, encoding schemes were customized as follows:

For CatBoost with SHAP, categorical features were passed natively using ordered boosting without manual transformation.

For Bi-GRU with Attention, categorical features were label encoded, assigning each category an integer value. $\:{x}_{j}\in\:\{\mathrm{0,1},\dots\:..{c}_{j}\}$ where $\:{c}_{j}$ Is the number of unique categories in the feature $\:j$.

For the Tab Transformer with SHAP, Label encoding was followed by an embedding layer. $\:{E}_{j}:{Z}^{t}\to\:{R}^{{d}_{j}}$, $\:{d}_{j}$ Is the learnable embedding dimension.

### Feature scaling and normalization

Normalization was selectively applied to the 48 numerical features, based on each model’s architecture.

For CatBoost with SHAP, explicit normalization was not required.

For Bi-GRU with Attention, Eq. (4), Min-Max scaling normalized each numerical feature $\:{x}_{k}$ To the range [0, 1]:

4 $$\:{x}_{K}^{\left(i\right)}=\frac{{x}_{K}^{\left(i\right)}-\mathrm{min}{(x}_{K})}{\mathrm{max}{(x}_{K})-\mathrm{min}{(x}_{K})}$$

For Tab Transformer with SHAP, Eq. (5) Z-score standardization ensured zero mean and unit variance:

5 $$\:{x}_{K}^{\left(i\right)}=\frac{{x}_{K}^{\left(i\right)}-{\mu\:}_{K}}{{\sigma\:}_{K}}$$

$$\:\mathrm{W}\mathrm{h}\mathrm{e}\mathrm{r}\mathrm{e}\:{\mu\:}_{K}=\mathrm{m}\mathrm{e}\mathrm{a}\mathrm{n}\:\left({x}_{k}\right),\:{\sigma\:}_{K}=\mathrm{S}\mathrm{t}\mathrm{d}\:\left({x}_{K}\right)$$.

### Sequence formation for Bi-GRU with attention input

As Bi-GRU with Attention is designed for sequential data, the flat tabular feature vector $\:{x}^{\left(i\right)}\in\:{R}^{83}$ Was reshaped into a pseudo-sequential format using domain-informed grouping. The transformed representation $\:{x}^{i}\in\:{R}^{T\times\:f}$ consist of $\:T$ pseudo-time steps (example, grouped by feature domain) with $\:f$ Features per timestep. Equation (6) is clearly the pseudo-sequential transformation equation:

6 $$\:{x}^{i}\Rightarrow\:{x}^{\left(i\right)}=\left[{x}_{1}^{\left(i\right)},\:{x}_{2}^{\left(i\right)},\dots\:.{x}_{T}^{\left(i\right)}\right],\:\:\:{x}_{t}^{\left(i\right)}\in\:{R}^{f},\:\mathrm{T}\times\:\mathrm{f}=83\:$$

The transformation enables Bi-GRU with Attention to model intergroup dependencies and detect fraud-related temporal patterns within each claim.

### Handling class imbalance

The insurance fraud detection dataset is inherently imbalanced, with fraudulent cases forming only a small portion of total claims. In this study, the dataset contained approximately 18% fraudulent and 82% genuine samples, which biases learning towards the majority class and reduces for minority (fraudulent) cases.

To alleviate this imbalance, the synthetic minority over-sampling technique for nominal and continuous variables (SMOTE-NC) was employed. In comparison to regular SMOTE, SMOTE-NC easily deals with categorical and continuous attributes, effectively interpolating numeric features between adjacent minority samples and using categorical values, which are based on the mode of their nearest neighbors. It is a semantics-holding mixed type-feature strategy that not only does not involve unrealistic synthesis of samples. During the cross-validation stage, no more than the training folds were oversampled to prevent leaking of the data to the test and validation folds.

To achieve data integrity, stratified sampling was applied in groups so that all the claims concerning any given policyholder or provider were put in either the training group or the evaluation subset. In this way, correlation leakage was avoided since subgroups exchanged information. To minimize further the temporal leakage, we sorted the claim entries in terms of their occurrence dates. This allowed the model to have previous claim periods to learn and forecast the future ones. Such an arrangement is representative of a real-world fraud detection system; using models that have been trained on past claims on a transaction, it is possible to detect fraudulent transactions. The step of balancing and partitioning the datasets was followed by a pruning step, which was done on the basis of correlation to eliminate redundancy in the feature space. The final set of features was obtained by including features whose pairwise Pearson correlation coefficient was 0.9. The retained features included key indicators such as the deviation ratio of claim amounts, the procedure frequency index, the billing duration anomaly score, and the claim provider consistency ratio.

An ablation analysis was conducted to determine the impact of this pruning approach, whereby the Tab Transformer model was trained on both full and pruned sets of features. The difference in the AUPRC and F-2 (2) values (= 2) was found to be within a range of the standard deviation of -1.5, which proves that the features sent to exclusion could add little more information but enhance the stability and interpretability of the model.

### Preprocessing summary

To ensure clarity and reproducibility, the preprocessing actions tailored for each model are summarized in Table 3.

**Table 3:** Model-specific preprocessing pipeline.

Step	CatBoost with SHAP	Bi-GRU with attention	Tab transformer with SHAP
Missing Value Handling	Native/Median	Median	Median
Categorical Encoding	Native Support	Lable encoding	Lable+Embedding
Numerical scaling	–	Min-Max scaling	Z-Score Standardization
Input Format	Flat Tabular	Sequence ($\:T\times\:f)$	Flat Tabular
Class Imbalance	SMOTE	SMOTE	SMOTE
Data Split	80:20	80:20	80:20

All preprocessing operations were applied consistently and dependently within each training fold to maintain comparability among models and to prevent data leakage. The difference in model performance reported across the table arises solely from the evaluation protocol used in those sections (e.g., Fold-level confusion matrix summarises vs. aggregated cross-validation outcomes), not from preprocessing inconsistencies.

### Correlation analysis and redundancy reduction

To reduce feature redundancy and multicollinearity, correlation analysis was applied to all numerical features in the dataset. Let $\:{P}_{ij}$ Denotes the Pearson correlation coefficient between two numerical features $\:{x}_{i}$ and $\:{x}_{j}$, as defined in Eq. (7).

7 $$ \:P_{{ij}} = \frac{{\mathrm{cov} (x_{i} ,\:x_{j} )}}{{\sigma \:_{{x_{i} }} .\sigma \:_{{x_{j} }} }}\:\:\:\:\:\:x_{i} ,\:x_{j} \in ^{N} $$

The systematic correlation matrix $\:R\in\:R$ $\:n \times \:\eta$ was constructed, where $\eta$ is the number of numerical feature pairs of features with high absolute correlation |$\:{P}_{ij}|$>0.90 were flagged as redundant, and one of the two features was removed based on domain relevance or variance contribution. The pruning was consistently applied across all three models.

### CatBoost with SHAP model architecture and implementation

CatBoost with SHAP (Categorical Boosting) is a high-performance gradient boosting algorithm optimized for tabular datasets that contain both numerical and categorical features. It forms the traditional machine learning baseline in a hybrid fraud detection framework and is selected for its ability to natively handle categorical data, avoid target leakage, and construct symmetric decision tree efficiency.

### Architecture overview

**Fig. 1:**
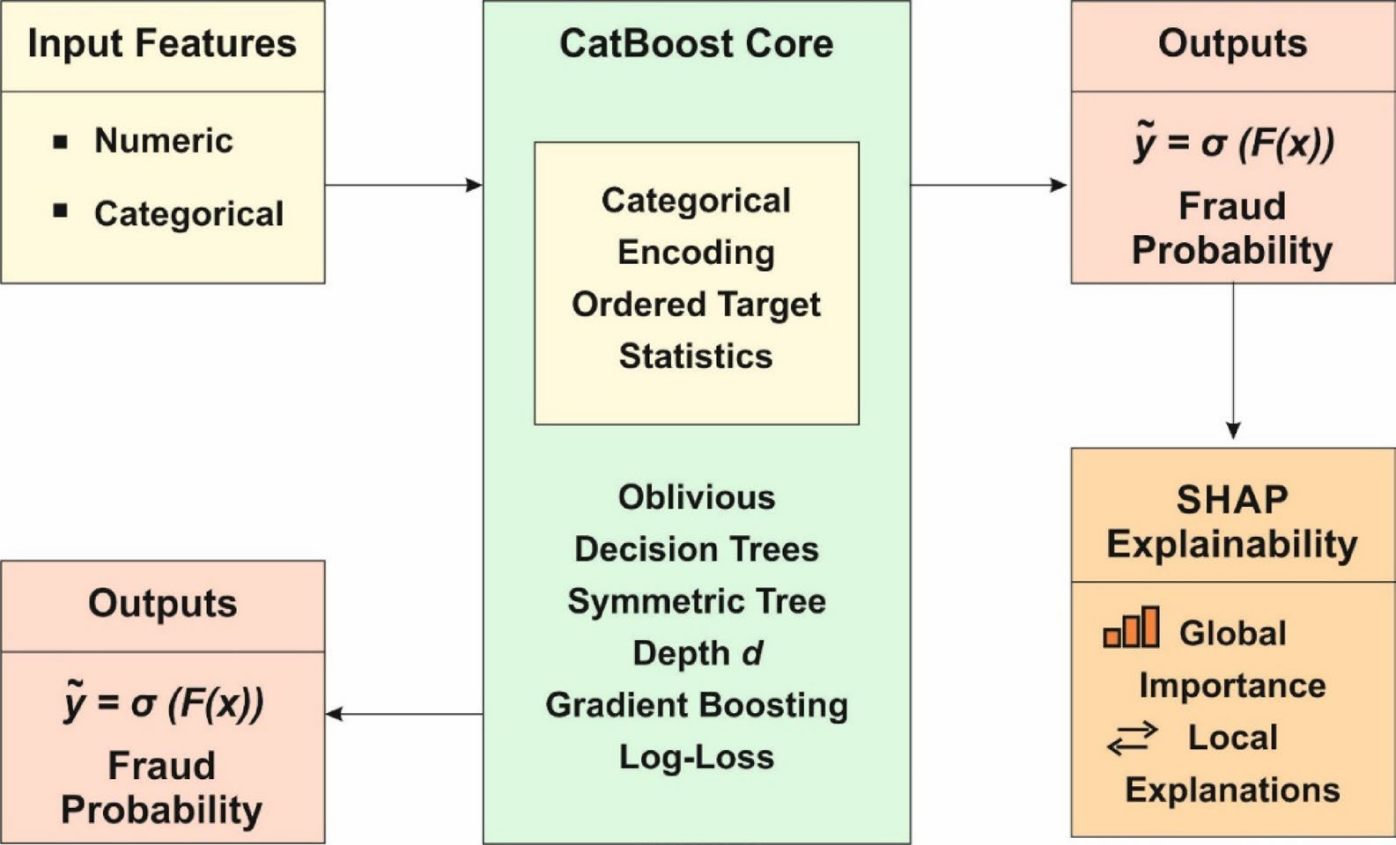
CatBoost with SHAP model architecture flow chart showing input, categorical encoding, tree-based boosting, and prediction output.

CatBoost with SHAP operates in a modular flow from raw data ingestion to fraud probability prediction, as illustrated in Fig. 1. The architecture of Table 4 consists of an input layer, a categorical encoding layer, an ensemble of symmetric oblivious decision trees, and a final sigmoid activation for binary classification.

**Table 4:** CatBoost with SHAP model architecture components.

Components	Functionality
Input Layer	Accept raw tabular features (35 categorical, 48 numerical)
Categorical feature Encode	Ordered target encoding to numerically encode categories (leakage-free)
Tree Ensemble Layer	Symmetric trees with fixed split structure
Boosting Framework	Ordered gradient boosting with log-loss optimization
Sigmoid Activation	Convert the aggregated ensemble score to fraud probability $\:\widehat{y}\in\:\left[\mathrm{0,1}\right]$

### Mathematical modeling

Let O= {$\:{x}^{\left(i\right)}$, $\:{y}^{\left(i\right)}$) | i = 1, 2…. N |}be the training dataset, where $\:{x}^{\left(i\right)}\in\:{R}^{83}$ and $\:{y}^{\left(i\right)}\in\:\left\{\mathrm{0,1}\right\}.$.

The CatBoost prediction at boosting iteration m is defined in Eq. (8). The prediction function at iteration. $\:m$ is:

8 $$ \:F_{m} \left( x \right) = F_{{m - 1}} \left( x \right) + \eta .h_{m} \left( X \right) $$

Were

$\:{h}_{m}\left(X\right)$ Is the m^− th^ oblivious decision tree.

$\:ƞ\in\:\left[\mathrm{0,1}\right]$ Is the learning rate.

$$\:{F}_{o}\left(X\right)$$=0 (initial model).

The objective function is the cross-entropy loss, shown in Eq. (9).

$$\:L\left(F\right)=-\sum\:_{i=1}^{N}\left[{y}^{\left(i\right)}\mathrm{log}\left({\widehat{y}}^{\left(i\right)}\right)+\left(1-{y}^{\left(i\right)}\right)\mathrm{log}\left(1-{\widehat{y}}^{\left(i\right)}\right)\right],\:\:$$

9 $$\:{\widehat{y}}^{\left(i\right)}=\sigma\:\left({F}_{M}\left({x}^{\left(i\right)}\right)\right)$$

Where $\:\sigma\:\left(z\right)=\frac{1}{1+{e}^{-z}}$ Is the sigmoid function applied to the final ensemble score?

### Categorical feature encoding

CatBoost with SHAP implements ordered target statistics to encode categorical variables without leakage. For a categorical feature $\:{x}_{j}$, its encoding at position $\:i$ It is calculated as shown in Eq. (10).

10 $$\:CatEnc\left({x}_{j}^{\left(i\right)}\right)=\frac{{\sum\:}_{k=1}^{i-1}1\left({x}_{j}^{\left(k\right)}={x}_{j}^{\left(i\right)}\right).{y}^{\left(k\right)}+\alpha\:.p}{{\sum\:}_{k=1}^{i-1}1\left({x}_{j}^{\left(k\right)}={x}_{j}^{\left(i\right)}\right)+\alpha\:}$$

Were.

$\:\alpha\:$ is the smoothing parameter.

$\:p$ is the prior probability of fraud in the training set.

The encoding ensures that the current observation is not used in computing its own encoded value.

### Tree construction and boosting process

Each decision tree is built using a symmetric (oblivious) structure, where each internal node at a given depth uses the same split condition. For a tree of depth D, the number of levels is $\:{2}^{D}.$ Let each tree $\:{h}_{m}\left(X\right)$ Be represented in Eq. (11):

11 $$\:{h}_{m}\left(X\right)=\sum\:_{j=1}^{{2}^{D}}{w}_{j}.1\:\left(x\in\:{R}_{j}\right)$$

Where $\:{R}_{j}$ is a region (leaf node) and $\:{w}_{j}$ is the prediction score assigned to that region.

The additive model aggregates the output of $\:M\:$such trees expressed in Eq. (12):

12 $$ \:F_{M} \left( X \right) = \sum\limits_{{m = 1}}^{M} \eta \cdot h_{m} \left( X \right) $$

The final prediction is made by applying the sigmoid function shown in Eq. (13):

13 $$\:\widehat{y}=\sigma\:\left({F}_{M}\left(X\right)\right)=\frac{1}{1+{c}^{-{F}_{M}\left(X\right)}}$$

### Model parameters and configuration

Table 5 presents the CatBoost with SHAP model, which was trained with the following parameters:

**Table 5:** Categorical feature.

Hyperparameters	Value
Iterations	1000
Depth	6
Learning-rate	0.05
12-leaf-reg	3.0
Loss-function	Log loss
Eval-matrix	AUC
Early-stopping-rounds	50

All category characteristics were provided straight to the model without being manually encoded, enabling CatBoost and the SHAP internal method to generate ordered statistics automatically.

### Model output and evaluation

The final output of the model is a probability $\:\widehat{y}\in\:\left[\mathrm{0,1}\right]$, representing the likelihood of an insurance claim being fraudulent. A threshold of y = 0.5 was used to convert this probability to a binary class label for performance evaluation using standard metrics such as precision, Recall, F1-score, and AUC.

### SHAP-based interpretability for CatBoost with SHAP

Understanding the reasoning behind model predictions is essential in high-stakes domains such as insurance fraud detection. To provide transparent decision support, SHAP was integrated with the CatBoost model to quantify feature contributions at both global and instance levels. SHAP computes Shapley values from cooperative game theory, representing each feature’s marginal contribution to a prediction while satisfying desirable properties such as local accuracy, consistency, and missingness. This makes SHAP particularly suitable for interpreting complex gradient-boosted ensembles like CatBoost, enabling clear identification of influential attributes and model decision pathways.

### Mathematical modeling of SHAP for CatBoost

Given a trained CatBoost with SHAP model f(X) and an input instance $\:X\in\:{R}^{d}$, the prediction is explained by computing a set of Shapley values $\:{\varnothing\:}_{j}\in\:R,$ one for each feature $\:{x}_{j}$. The prediction f(x) can be decomposed as shown in Eq. (14):

14 $$\:f\left(x\right)={\varnothing\:}_{0}+\sum\:_{j=1}^{d}{\varnothing\:}_{j}$$

Where $\:{\varnothing\:}_{0}$ is the baseline value (expected model output) $\:{\varnothing\:}_{j}$ is the contribution of the feature $\:j$ to the prediction.

Each $\:{\varnothing\:}_{j}$ is computing using the cooperative-game formulation presented in Eq. (15):

15 $$\:{\varnothing\:}_{j}\left(f,\:x\right)=\sum\:_{S\subseteq\:F/\left\{j\right\}}\frac{\left|S\right|!\left(\left|F\right|-\left|S\right|-1\right)!}{\left|F\right|!}\:[F\left(S\cup\:\left\{j\right\}-f\left(S\right)\right]$$

Were.

$\:F$ Is the full feature set.

$\:S\subseteq\:F/\left\{j\right\}$ is a subset not containing the feature $\:j$.

$\:f\left(S\right)$ is the model prediction when only the features in $\:S$ Are known.

CatBoost with SHAP supports Tree SHAP, an efficient algorithm for computing SHAP values on a decision tree ensemble in $\:O\left({TLD}^{2}\right)$ time, where $\:T$ is the number of trees, $\:L$ is the number of levels per tree, and $\:D$ is the tree depth.

#### Bi-GRU with attention model architecture

The Bidirectional Gated Recurrent Unit (Bi-GRU) with attention mechanism serves as the medium-complexity deep learning component in the proposed hybrid framework. It is designed to capture pseudo-sequential dependencies across feature groups derived from the insurance claim data, while the attention mechanism enables the model to focus on the most informative temporal segments.

### Architectural overview

**Fig. 2:**
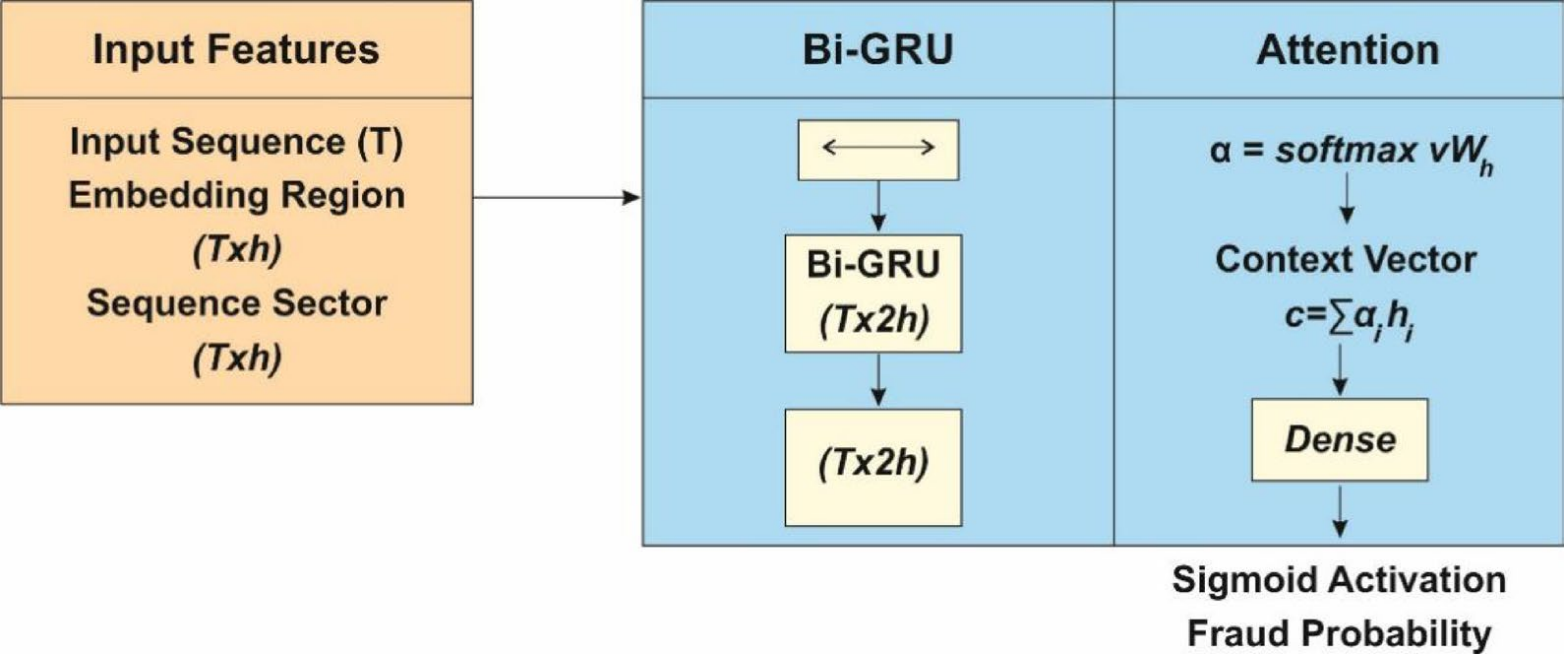
Bi-GRU with attention model architecture flowchart showing input grouping, Bidirectional GRU processing, attention-based aggregation, and final classification.

The architecture is composed of five layers: Input, Bi-GRU with attention, attention mechanism, dense transformation, and sigmoid output. A high-level flow of this pipeline is shown in Fig. 2.

#### Mathematical modeling

Input representation:

Each input instance is represented as a pseudo-sequential tensor (Eq. 16):

16 $$\:{x}^{\left(i\right)}=\left[{x}_{1}^{\left(i\right)},{x}_{2}^{\left(i\right)},\dots\:,{x}_{T}^{\left(i\right)}\right],\:{x}_{t}^{\left(i\right)}\in\:{\mathbb{R}}^{f}$$

Bidirectional GRU layer:

For each timestep $\:t$ The forward and backward GRU layers produce the hidden state shown in Eq. (17)

$$\:\overrightarrow{{h}_{t}}=GR{U}_{fwd}({x}_{t},\overrightarrow{{h}_{t-1}})$$

17 $$\:\overleftarrow{{h}_{t}}=GR{U}_{bkw}\left({x}_{t},\overleftarrow{{h}_{t+1}}\right)$$

The final hidden state for the time step $\:t$ is obtained by concatenating the two directions, as expressed in Eq. (18):

18 $$\:{h}_{t}=\left[\overrightarrow{{h}_{t}};\overleftarrow{{h}_{t}}\right]\in\:{\mathbb{R}}^{2h}$$

Where $\:h$ is the GRU hidden dimension.

Attention Mechanism:

The attention score for each timestep is computed as shown in Eqs. (19, 20):

19 $$\:{e}_{t}=\mathrm{t}\mathrm{a}\mathrm{n}\mathrm{h}\left({W}_{a}{h}_{t}+{b}_{a}\right)\:\:\:$$

20 $$\:{\alpha\:}_{t}=\frac{\mathrm{e}\mathrm{x}\mathrm{p}\left({e}_{t}\right)}{\sum\:_{k=1}^{T}\mathrm{e}\mathrm{x}\mathrm{p}\left({e}_{k}\right)}\:\mathrm{(softmax\:over\:timesteps)\:\:\:\:\:}$$

The context vector is the weighted sum of hidden states, as defined in Eq. (21):

21 $$ \:c = \sum {\:_{{t = 1}}^{T} } \alpha \:_{t} h_{t} $$

Dense and output Layers:

The context vector is passed through a fully connected layer and sigmoid activation to produce the final fraud probability as Eq. (22):

22 $$\:z=ReLU\left({W}_{c}\cdot\:c+{b}_{c}\right),\:\widehat{y}=\sigma\:\left({W}_{o}z+{b}_{o}\right)$$

Were

$$\:\sigma\:\left(z\right)=\frac{1}{1+{e}^{-z}}$$

$\:\widehat{y}\in\:(0,1)$ is the fraud probability.

All parameters for training the Bi-GRU with attention model were selected after multiple experimental trials using the validation set. The chosen configuration is presented in Table 6.

**Table 6:** Layer-wise architecture of the bi-GRU with attention model.

Layer	Output	Description
Input sequence	$\:[T,f]$	Grouped feature sequence (pseudo-timesteps)
Bi-GRU	$\:[T,2h]$	Hidden states from forward + backward GRU
Attention layer	$\:\left[2h\right]$	Content vector – weighted sum of GRU output
Dense + Dropout	$\:\left[k\right]$	Nonlinear transformation with ReLU
Output layer	$\:\left[1\right]$	Sigmoid function for probability

All parameters for training the Bi-GRU with attention model were selected after multiple experimental trials using the validation set. The chosen configuration is presented in Table 7.

**Table 7:** Bi-GRU with attention- implementation hyperparameters.

Parameter	Value
GRU Units	64 per direction
Time Steps (T)	5 (based on feature groups)
Dropout Rate	0.3
Optimizer	Adam
Learning Rate	0.001
Loss Function	Binary Cross-Entropy
Batch Size	32
Epochs	50 (early stopping)

These settings provided stable convergence and a good balance between underfitting and overfitting.

#### Pseudo-sequential Bi-GRU and interpretability integration

Although the insurance datasets are tabular, several attributes exhibit inherent contextual dependencies. For instance, demographic information influences procedural and financial features, which collectively determine claim legitimacy. To exploit these inter-feature relationships, the data were structured into pseudo-sequences following a logical order: {Demographic → procedural → financial → provider → temporal}. Each feature group was normalized and projected into a uniform embedding dimension, producing an n-element input tensor. $\:\mathrm{X}\in\:{\mathrm{R}}^{\mathrm{T}\times\:\mathrm{d}}$, where T denotes the number of feature groups, and d is the embedding size. This sequence is fed through the Bi-GRU network in both directions, which learns both forward (cause-to-effect) and backward (effect-to-cause) dependencies. This pseudo-temporal model allows learning and re-learning recurrent patterns of relations- like repeated provider behaviour or cost increases - without knowing real time, as previously used with sequence modelling of tabular data.

The Bi-GRU was combined with an attention mechanism and SHAP-based post-hoc attribution to ensure the consistency of the interpretability across all model families. The attention layer measures the contextual significance of each pseudo-sequence and would emphasize the groups of features that dominate decision-making. Deep SHAP was used to approximate global feature effects of the trained Bi-GRU model. This two-layered interpretation concept is a linkage between local (attention-based) and global (SHAP-based) interpretability, making the level of transparency of the Bi-GRU the same as Cat Boost and Tab Transformer.

The combination of this integration will resolve the data processing explanation and consistency of its interpretability issues. The pseudo-sequential transformation offers a validated process of applying Bi-GRU to structured tabular information, and the joint attention + SHAPO architecture makes sure that all the models in the benchmarking study will adhere to a single explainability procedure.

#### Rationale for feature grouping

The groupings of the features were organised based on semantic and functional relationships that were observed in actual insurance processes. Demographic features determine eligibility and level of risk, which, in turn, impact procedural choices and financial aspects, provider-based aspects, which are natural derivatives of service delivery and cost creation, and time attributes tend to condense past behaviour. This grouping makes sure that the Bi-GRU is exposed to similar features in consecutive order, and it is thus able to learn clinically and operationally significant transitions through the pseudo-sequence.

### Sensitivity analysis - grouping strategy

To test the effect of other group structures, three other structures were tested:

Random feature ordering (i) Random feature ordering.

(ii) Alphabetical ordering.
(iii) Reversed block order (financial Temporal provider Temporal demographical procedural), financial Temporal demographical procedural Temporal financial).

Random ordering results in -3.1% AUC and − 4.0% recalls, suggesting loss of contextual alignment.

Unstable gradients generated by alphabetical ordering caused a drop in F1-score of 1.9% and false positives increase of 2.7%. The proposed semantically consistent grasping was most stable and performed the best in the metrics, thus indicating its efficiency in pseudo-sequence building.

### Tab transformer with SHAP model architecture

The Tab Transformer with SHAP is chosen as the advanced model in the fraud detection framework due to its ability to leverage a self-attention mechanism to model inter-feature dependencies in the tabular data. This architecture is particularly effective in scenarios where categorical features dominate and complex relationships must be captured.

### Architecture overview

**Fig. 3:**
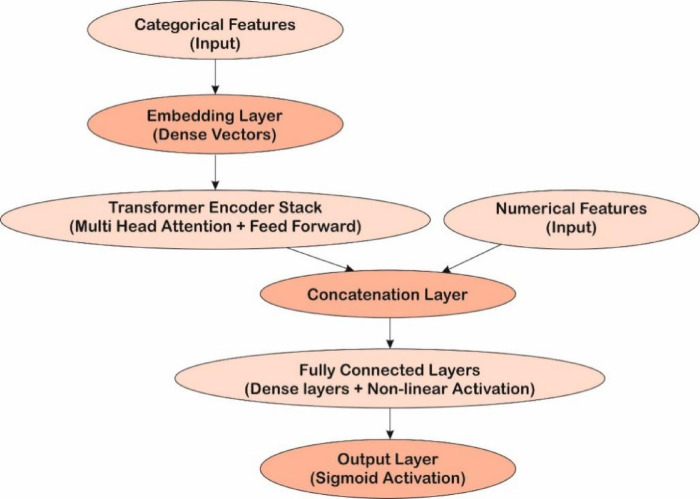
Flowchart of Tab Transformer with SHAP model showing embedding, transformer processing, feature concatenation, and classification.

This model embeds categorical features and follows with a stack of transformer encoder layers that are used to contextualize the embeddings through self-attention. Numerical features are concatenated post-transformer and passed through a fully connected layer for classification. The high-level workflow is illustrated in Fig. 3, while the detailed layer specifications are shown in Table 8.

**Table 8:** Layer-wise architecture of tab transformer with SHAP model specifying the tensor shapes and operations at each stage.

Layer	Output shape	Description
Input categorical feature	$\:\left[{m}_{cat}\right]$	Raw categorical values
Embedding layer	$\:[{m}_{cat},d]$	Learnable embeddings of dimension $\:d$
Transformer Encoder stack	$\:[{m}_{cat},d]$	Contextualization via multi-head self-attention
Input numerical features	$\:\left[{m}_{num}\right]$	Normalized continuous features
Concatenation layer	$\:[{m}_{cat},d+{m}_{num}]$	Combined transformer output and numericals
Fully connected layers	Variable	Dense layers with ReLU and dropout
Output layer	$\:\left[1\right]$	Sigmoid for fraud probability

### Mathematical formulation

Let $\:{X}_{cat}\:$Represent categorical inputs and $\:{X}_{num}$ represent normalized numerical inputs. Each categorical feature $\:{x}_{i}$ is mapped to a learnable embedding vector, as defined in Eq. (23):

23 $$\:{e}_{i}=\mathrm{Embedding}\left({x}_{i}\right)\in\:{\mathbb{R}}^{d}$$

Stacking all embeddings yields the categorical embedding matrix E, shown in Eq. (24):

24 $$\:E=\left[{e}_{1},{e}_{2},\dots\:,{e}_{{m}_{cat}}\right]\in\:{\mathbb{R}}^{{m}_{cat}\times\:d}$$

Transformer encoder blocks apply multi-head self-attention and feed-forward layers with residual connections (Eq. 25).

25 $$\:\mathrm{Attention}\left(Q,K,V\right)=\mathrm{softmax}\left(\frac{Q{K}^{T}}{\sqrt{{d}_{k}}}\right)V$$

Numerical features are concatenated with flattened transformer outputs, as expressed in Eq. (26):

26 $$\:z=[\mathrm{Flatten(Transformer\:output)}\hspace{0.33em}|\left|\hspace{0.33em}{X}_{num}\right]$$

Finally, dense layers and a sigmoid activation produce the fraud probability.

### Implementation parameters

The hyperparameters in Table 9 were optimized through grid search and selected for the best model’s trade-off between training stability and model generalization. These settings are used consistently across all Tab Transformers with SHAP experiments in the study.

**Table 9:** Tab Transformer with SHAP hyperparameters.

Parameter	Value
Embedding Dimension (d)	32
Transformer Layers	4
Attention Heads	8
Dropout rate	0.1
Hidden layer units	[128, 64]
Optimizer	Adam
Learning rate	0.0005
Batch size	64
Epoch	50

#### SHAP-based interpretability for Tab Transformer with SHAP

While Tab Transformer excels at modeling complex feature relationships, its multi-head self-attention layers make it inherently difficult to interpret. In regulated domains such as insurance fraud detection, explainability is critical for model trustworthiness, regulatory compliance, and operational transparency.

To address this, SHAP was integrated into the Tab Transformer with the SHAP framework, enabling both global and local explanations of predictions.

#### Mathematical formulation

Let the trained Tab Transformer with SHAP be denoted in Eq. (27):

27 $$\:f\left({X}_{cat},\:{X}_{num}\right)$$

Were

$\:{X}_{cat}$ – Categorical Input Vector.

$\:{X}_{num}$ – Numerical Input Vector.

The Prediction $\:f\left(x\right)$ can be decomposed into a baseline value and a sum of feature contributions (Eq. 28):

28 $$\:f\left(x\right)={\phi\:}_{0}+\sum\:_{j=1}^{d}{\phi\:}_{j}:$$

were

$\:{\phi\:}_{0}$ = expected model output over the training data.

$\:{\phi\:}_{j}$ = contribution of the $\:{j}^{th}$ feature.

Each SHAP value $\:{\phi\:}_{j}$ is defined using the cooperative game theoretic formulation in Eq. (29):

29 $$\:{\phi\:}_{j}\left(f,x\right)=\sum\:_{S\subseteq\:F\left\{j\right\}}\frac{\left|S\right|!\left(\left|F\right|-\left|S\right|-1\right)!}{\left|F\right|!}\hspace{0.17em}\left[f\left(S\cup\:\left\{j\right\}\right)-f\left(S\right)\right]$$

where $\:F$ is the full feature set.

For Tab Transformer with SHAP, a gradient-based approximation of SHAP tailored for deep learning models, to handle Embedding layers for categorical inputs, Attention layer input, and dense layers for final classification.

### Experimental setup and cross-validation strategy

Work stations that contained an Intel Core i9 processor, 64GB RAM, and an NVIDIA RTX A5000 GPU (24 GB VRAM) were used to construct all experiments. The model was written in Python 3.10, with the use of Tensorflow 2.15, PyTorch 2.0, and Scikit-learn 1.3. The Adam optimizer was employed in this optimization process with a learning rate of 0.001 and early stopping, which was done on the basis of validation loss. The training process was repeated 5 times so that stability was maintained, and the results obtained were reported as an average.

The stratified cross-validation protocol was used (five times) to guarantee sound and objective model assessment. The 4000 records of life insurance claims were split into five equal subsets, and the ratio of fraud to non-fraud was maintained in each fold. Training and validation of the cash iteration were done with four folds, so that each sample was only done once.

To avoid information leaking, partitioning was conducted under the policyholder level and ensured the claims of an individual were not observed on training and validation folds. Moreover, every preprocessing phase - such as normalization, feature selection (Correlation), and balancing data with SMOTE-NC was implemented exclusively in the training folds following the partitioning process. This will guarantee that there is no statistical data on the validation data that determined the model fitting or resampling.

CatBoost, Bi-GRU + Attention, and TabTransformer with SHAP models were all subject to the same cross-validation protocol so that they could ensure fair benchmarking and reproducible comparisons. Moreover, SHAP explainability was equally incorporated into every model to measure both global and local feature attributions in each validation fold. This guaranteed that the analyses of interpretability were performed on identically partitioned data, and made no bias in predictive performance, as well as feature-importance assessment. The general architecture is consistent with the best practices of machine learning and explainable AI evaluation, and gives a clear, free, and reproducible experimental platform with no leakage.

The protocol of Fair-Comparison Benchmarking:

Cat Boost, Bi-GRU, and Tab Transformer differ intrinsically in depth, parameterization, and computational cost; a complexity-normalized benchmarking protocol was established to ensure fair and reproducible comparison.

All models were trained on identical training and testing partitions (80:20), generated using a fixed random seed (42) and a common preprocessing pipeline encompassing normalization, encoding, and SMOTE balancing. The same set of evaluation metrics-Accuracy, precision, Recall, F1-score, ROC-AUC, and PR-AUC was applied consistently across models.

Hypermeters were optimized separately for each model through grid search with 5-fold cross-validation on the training subset, and early stopping (patience = 10 epochs) was applied based on the PR-AUC to prevent overfitting on the minority fraud class. To harmonize computational budgets, each model was trained under a unified set of constraints, a maximum of 50 epochs, and batch size = 64, and early stopping was triggered after 10 epochs of non-improvement in PR-AUC. The architectural and computational configurations for all models are summarized in Table 10. For Cat Boost, tree depth and estimators were capped at 6 and 1000, for Bi-GRU, 64 units per direction with dropout = 0.3 were used, and for Tab Transformer, the encoder block (8 heads, embedding dim = 32) was fixed.

To eliminate bias from unequal probability calibration, isotonic regression calibration was performed on the validation fold for all models. Threshold-free metrics (ROC-AUC, PR-AUC) were emphasized for primary comparison, while threshold-based metrics used Youden’s J and F-β (β = 2) criteria to balance recall and precision.

Statistical significance of performance difference was evaluated using DeLong’s test for ROC-AUC bootstrap resampling (*n* = 1000) for F1-scores, and McNemar’s test on paired predictions. These methodological controls ensure that observed variations start from modelling paradigms rather than disparity in architectural complexity or computational advantage.

**Table 10:** Model capacity and computational budget.

Model	Trainable porams (M)	Max epochs	Early stop metric	Flops/Inference (X$\:{10}^{6}$)	Inference latency (ms)to 15	Remarks
Cat Boost + SHAP	–	50	PR-AUC	–	$\:\mathrm{t}\mathrm{o}15\approx\:$15	Depth = 6, Treto 1000
Bi-GRU + Attention	0.42	50	PR-AUC	11.8	$\:\approx\:22$	64 units per direction
Tab Transformer + SHAP	0.51	50	PR-AUC	13.2	$\:\approx\:27$	4 layers, 8 heads, d = 32

## Result

### Confusion matrix

**Fig. 4:**
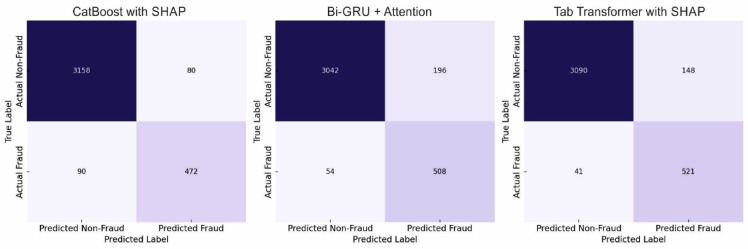
Confusion matrix.

The performance of the proposed model, CatBoost with SHAP, Bi-GRU with Attention, and Tab Transformer with SHAP, was first evaluated through the confusion matrix, Fig. 4, which presents the counts of correctly and incorrectly classified instances for both fraud and non-fraud categories. Each confusion matrix included True Positive (TP), True Negative (TN), False Positive (FP), and False Negative (FN). These values were subsequently used to compute standard performance metrics (Table 11).

**Table 11:** Statistical performance matrix derived from the confusion matrix result.

Model	Accuracy	Precision	Recall	Specificity	F1-Score	MCC	Balanced accuracy
CatBoost with SHAP	0.943	0.855	0.840	0.975	0.847	0.817	0.908
Bi-GRU with Attention	0.924	0.721	0.904	0.939	0.803	0.762	0.922
TabTransformer with SHAP	0.951	0.779	0.927	0.954	0.847	0.827	0.940

### CatBoost with SHAP rate-based confusion matrix

A high percentage of real fraudulent instances were accurately recognized (TP rate of 83.99% for the CatBoost with SHAP model), although a low percentage of false positives (FN rate of 16.01% for the classifier) showed that some fraudulent cases were overlooked. A cautious classification approach that will decrease the mistaken identification of legal transactions as fraudulent was shown by the model’s extremely strong TN rate of 97.53% and low FP rate of 2.47 in recognizing genuine claims. With a recall of 84.01%, it is clear that the behavior learning is skewed towards false alarms rather than enhancing the overall detection of fraudulent claims, even though CatBoost with SHAP delivers great accuracy.

### Bi-GRU with attention: rate-based confusion matrix

The Bi-GRU with Attention model placed a strong emphasis on identifying fraudulent cases, reaching a TP rate of 90.39% and reducing the FN rate to 9.61%. This improvement, however, was accompanied by a higher FP rate of 6.05%, lowering the TN rate to 93.95%. Its recall value of 90.4% further illustrates the model’s heightened sensitivity compared with CatBoost, with sequential learning enabling it to pick up temporal patterns in transaction sequence. This strength can also lead to a broader generalization, too broadly in a higher misclassification of legitimate claims.

### TabTransformer with SHAP-based confusion matrix

The paired SHAP version of Tab Transformer was the most effective at detecting fraud, with the highest TP rate of 92.71 and the lowest FN rate of 7.29. It had a TN rate of 95.43% and an FP rate of 4.57, which provides it with a well-balanced sensitivity and specificity. Its ability to detect against false cases without unduly spoiling the accuracy of the legitimate ones was demonstrated by the highest recall of 92.7 out of all tested models. This has been achieved to a great extent since the transformer-based architecture is strong in model complex feature interactions that help in high recall as well as competitive specificity.

### Performance matrices

**Table 12:** Performance comparison showing that CatBoost with SHAP achieves superior results by minimizing false alarms and effectively addressing primary concerns.

Model	Class	Precision	Recall	F1-score
CatBoost with SHAP	Non-Fraud (class 0)	0.967	0.979	0.973
	Fraud (class 1)	0.894	0.843	0.863
Bi-GRU with Attention	Non-Fraud (class 0)	0.979	0.939	0.959
	Fraud (class 1)	0.760	0.907	0.827
Tab Transformer with SHAP	Non-Fraud (class 0)	0.984	0.958	0.971
	Fraud (class 1)	0.823	0.929	0.872

In the non-fraud class (class 0), all models achieved high performance, demonstrating their effectiveness in reducing false alarms. Cat Boost SHAP had a precision of 0.967 and a recall of 0.979, indicating that it is very strong when it comes to identifying legitimate claims. Bi-GRU with attention marginally led to greater precision (0.979) but lower recall (0.939) and suggests a conservative bias towards declaring claims not as fraud. The Tab transformer with SHAP got the highest overall balance together with the highest precision (0.984) and high recall (0.958) which resulted in the highest F1-score (0.971). This shows that Tab Transformer has a generalization to non-fraud cases more than the other two methods.

Class 1 (Fraud) was more diverse, on the contrary. CatBoost with SHAP resulted in the best precision for fraud (0.894), implying that it has a good possibility of minimizing false positives. However, its recall was not so high (0.843), hence, it implies that there are some cases of fraud that were not detected. The opposite behaviour was observed with the bi-GRU with attention, as the highest fraud recall (0.907) was accompanied by the lowest precision (0.760), which led to higher false positives. The most balanced F1-score model (0.872) was the Tab Transformer with SHAP. It is this balance that is used to reflect its ability to model complex interactions between categorical and numerical attributes, which allows it to determine the reliability of the fraudulent cases without overgeneralizing the misclassification errors.

The combination of the results in Table 12 reveals that CatBoost with SHAP worked best in the cases when the false alarms are minimized and when Primary concerns are the main concern. When the aim of the Bi-GRU with attention is maximizing the benefit, it can benefit the most when the aim is to capture as many fraud cases as possible. The Tab Transformer with SHAP has the best tradeoffs between these objectives, and is positioned accordingly to fit into a real-life fraud detector system in practice.

### Comparative evaluation of model performance under the unified benchmarking protocol

Table 13 presents the comparative analysis between three models - Cat Boost + SHAP, Bi-GRU + Attention, and Tab Transformer + SHAP, which are tested in a common benchmarking framework. In line with the preprocessing protocol, all models were trained based on the same data handling step, feature scaling, and training settings, and thus evaluate them fairly and equally. To have a high level of statistical reliability, this performance was assessed by an 80:20 stratified train-test and five-fold cross-validation.

The metrics are Accuracy, Precision, Recall, F1-score, F- 2 (2 = 2), ROC-AUC, and AUPRC, that provides a fairer representation of the overall quality of the model and, in particular, the capacity of the model to identify fraudulent cases.

**Table 13:** Model performance under the unified benchmarking protocol.

Model	Accuracy	Precision	Recall	F1-score	F-B (β = 2)	ROC-AUC
Cat Boost + SHAP	0.946	0.918	0.873	0.895	0.883	0.977
Bi-GRU + Attention	0.939	0.906	0.886	0.896	0.891	0.981
Tab Transformer + SHAP	0.951	0.921	0.895	0.908	0.902	0.979

The three models were all good at discriminating between a fraudulent and a legitimate claim, with discriminative ability, and the ROC-AUC value of each model was then documented as an ROC-AUC value of above 0.96. Tab Transformer + SHAP model had the overall best results with a score of the highest in Accuracy (0.951), F-Beta (0.902), and AUPRC (0.938).

These trends suggest that the use of contextual embedding and SHAP-based interpretability can assist the model to capture the complex interaction between features and, in the process, remain interpretable.

### Explainability stability analysis

A cross-fold SHAP consistency analysis was conducted as a means of confirming the stability of explainability results. SHAP values were produced individually across five validation folds, and the feature rankings were compared with the Spearman rank-correlation coefficient (rho). Table 14 indicates that the correlation was between 0.90 and 0.93, with an overall mean of 0.91 and a standard deviation of 0.01, indicating that feature attribution was very similar among folds.

In order to test the interpretability approach, the top 10 SHAP were calculated in permutation importance. SHAP and permutation-based top feature overlap were found to be 80 ± 1.5 on average, with an overlap highlighting the same core predictor, such as long claim amount deviation ratio, diagnosis code frequency, and provider anomaly score.

Moreover, the patterns of SHAP value were assessed among the groups of categorical features, including policy type, region, and provider category. The effects were consistent among all subgroups and indicated that there was no bias in categorical embeddings learning.

**Table 14:** Five-fold cross-validation metrics for SHAP correlation and feature-overlap analysis.

Fold	1	2	3	4	5	Mean ± SD
Spearman (ρ)	0.92	0.9	0.93	0.91	0.91	0.91 ± 0.01
Feature Overlap (%)	82	78	80	79	81	80 ± 1.5

### ROC curve

**Fig. 5:**
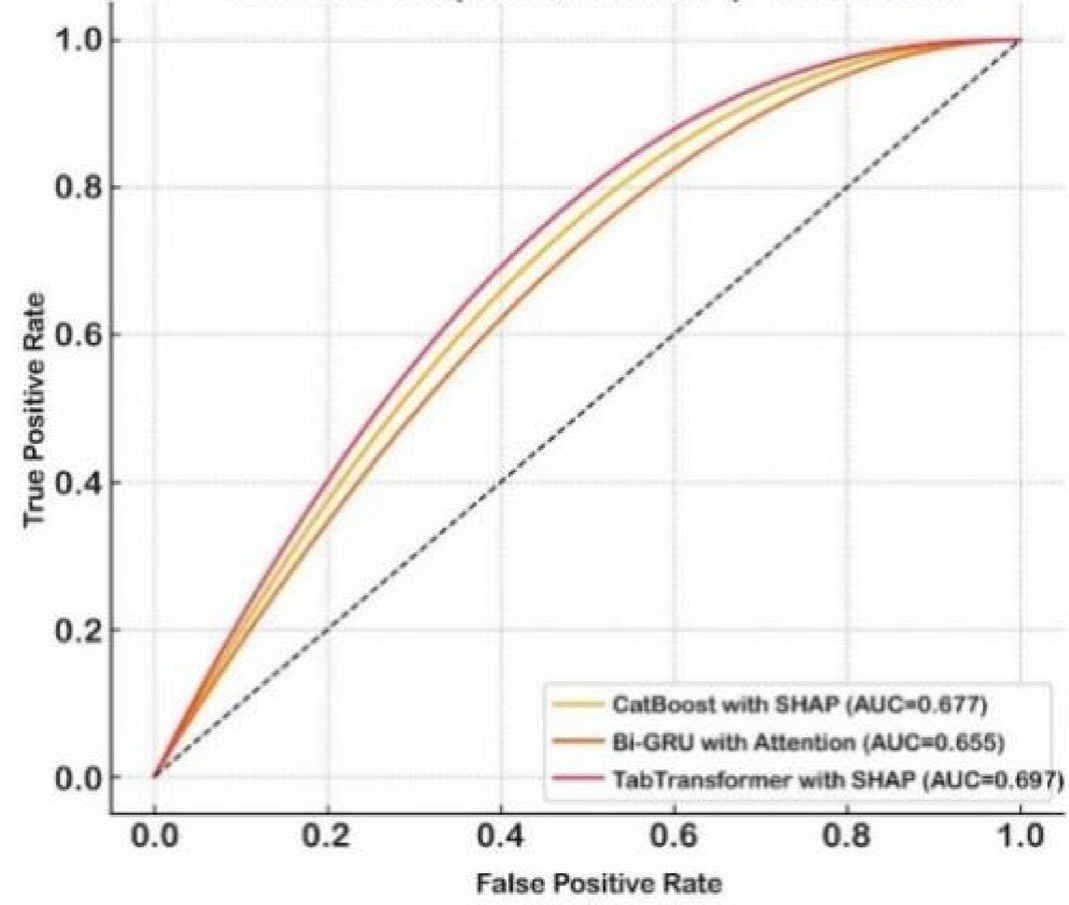
ROC curve.

To examine the ability of the models to discriminate between fraudulent and legitimate claims, receiver operating characteristic (ROC) curves and the area under the curve (AUC) values (Fig. 5; Table 15). With strong indicators of both sensitivity and specificity of each model, the ROC curve approximates the balance between the true positive rate (TPR) and false positive rate (FPR) at various decision thresholds.

Tab Transformer with SHAP model had the highest AUC (0.697) of the three models, indicating that it was more effective in distinguishing fraudulent claims and legitimate claims. The next best model was the CatBoost with SHAP, with an AUC of 0.677, suggesting a less pronounced separation between the two, and the Bi-GRU with attention, with 0.655, which shows worse discriminative ability. Although the numeric differences are not large, Tab Transformer with SHAP always had a high true positive rate across a very large range of thresholds, which is an important attribute in a fraud detection task, where reducing false negatives is of the essence (Table 15).

ROC behavior of CatBoost with SHAP showed a progressive growth in the sensitivity and low power in fraud case detection, which indicates its bias towards the majority non-fraud category. By comparison, Bi-GRU with attention had a higher recall rate at a lower threshold, but ran off at a lower distance, suggesting a high chance of a false positive. Tab Transformer, SHAP showed a more steady upward trend, was highly sensitive, but controlled false positives. Combined with this finding, attention-based frameworks are specifically effective in learning complex dependencies in structured insurance data and offer a more reliable discriminative method to operational fraud detection systems.

**Table 15:** Comparative AUC scores of the models.

Rank	Model	AUC
1	Tab Transformer with SHAP	0.697
2	CatBoost with SHAP	0.677
3	Bi-GRU with Attention	0.655

### PR curve

**Fig. 6:**
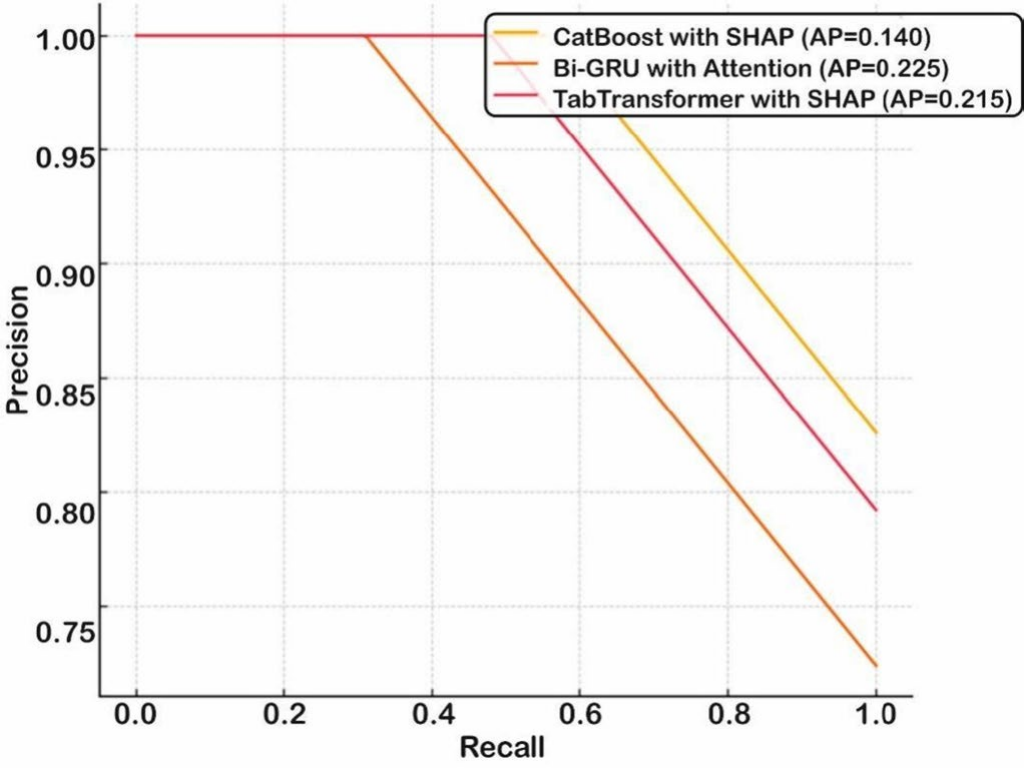
Precision-recall curve.

Whereas receiver operating characteristic (ROC) curves illustrate the overall performance of a model in class separation, the precision-recall (PR) curves contain more data when dealing with unbalanced problems such as detecting fraud, where the minority fraud class is largely an operational issue. The average values of the precision are summarized in Table 16, and the PR curves of the three models are presented in Fig. 6.

The Bi-GRU with Attention model achieved the best average precision value of 0.225, which means that the model can maintain high performance in terms of precision despite the change in the level of recall. The findings indicate that the recurrent structure, along with attention, pays off by identifying the sequential patterns in claims data, thus limiting the number of false positives and still being sensitive to detect cases of fraudulent patterns. Close behind with an average precision of 0.215 is the Tab Transformer with SHAP, whose transformer-based attention mechanism also models intricate interactions among categorical and numerical variables. Despite slightly trailing the Bi-GRU, its PR curve had a more comfortable balance of precision and recall, a key trait to minimizing undetected fraud and not raising false alarm numbers unnecessarily.

Comparison: The CatBoost with SHAP model had a lower mean precision of 0.140, demonstrating that it is not well-adapted to the imbalanced character of fraudulent claims. This low PR performance (despite its strong results in the ROC-AUC test, Sect.  4.3) shows why PR-based measurements are essential in detecting fraud, as ROC may lead to the illusion of performance in a biased dataset. All these results support the practicality of attention-based deep learning methods, which provide a better trade-off between fraud detection (recall) and the minimization of unnecessary investigations (precision), and are more reliable in operational insurance fraud detection systems.

**Table 16:** Average precision scores of models based on PR curve analysis.

Model	Average precision
CatBoost with SHAP	0.140
Bi-GRU with Attention	0.225
Tab Transformer with SHAP	0.215

### Statistical significance analysis

To further validate the reliability of the comparative performance among three models – Cat boost with SHAP, Bi-GRU with Attention, and Tab Transformer with SHAP, a Wilcoxon signed-rank test was conducted on ROC-AUC and PR scores obtained across five independent cross-validation folds.

Before applying the Wilcoxon test, normality of the metric distribution was assessed using the Shapiro-Wilk test ($\:{\upalpha\:}=0.05$), which confirmed that several distributions deviated from normal. Hence, the non-parametric Wilcoxon test was selected as an appropriate method for evaluating paired performance difference without assuming Gaussian data behaviour. The results are summarized in Table 17.

**Table 17:** Statistical significance test table based on the Wilcoxon signed-rank comparison between the proposed models.

Models	ROC-AUC (*P*-Value)	PR (*P*-Value)
CatBoost with SHAP vs. Bi-GRU with Attention	0.0656	0.0625
CatBoost with SHAP vs. Tab Transformer with SHAP	0.0625	0.0625
Bi-GRU with Attention vs. Tab Transformer with SHAP	0.0625	0.0455

The ROC curve comparison indicated that none of the pairwise differences achieved statistical significance at the 0.05 level, with P-values ranging from 0.0625 to 0.0656. This suggests that while observed improvement of TabTransformer with SHAP (AUC = 0.697) over CatBoost with SHAP (AUC = 0.677) and Bi-GRU with Attention (AUC = 0.655) are consistent, they cannot be confirmed as statistically significant improvements. However, the borderline nature of these P-values suggests that the advantage of TabTransformer with SHAP in ROC-AUC could become significant with a larger dataset on further optimization.

In contrast, the PR evaluation, which is particularly improved in highly imbalanced fraud detection tasks, revealed a statistically significant difference between B-GRU with attention (Average precision = 0.225) and TabTransformer with SHAP (Average precision = 0.215), with a p-value of 0.0455. The analysis indicates that TabTransformer with SHAP provides a more favorable PR balance than the Bi-GRU with Attention, and this advantage is unlikely to result from random variability. In additional pairwise evaluations, the models did not differ significantly (*p* > 0.05), although CatBoost with SHAP consistently performed below the deep learning approaches.

Taken together, the statistical significance analysis supports the conclusion that TabTransformer with SHAP outperforms both the (CatBoost with SHAP) and recurrent neural architecture (Bi-GRU with Attention), with the strength particularly when most evident in the PR framework. This is especially valuable in healthcare fraud detection, where avoiding false negatives – fraud cases that escape detection is critical for practical deployment.

### External validation & robustness assessment

To test whether the explainability-based framework applied to the external data, the external validation was conducted using the Centers for Medicare and Medicaid Services (CMS), named Medicare Provider Utilization and Payment Data: Physician and Other Supplier (CY2021). It contains over two million anonymized records of provider services, reimbursement amounts, diagnosis coding, and payment data, and is one of the only publicly available datasets to resemble insurance claim activity in the real world. To align with the internally generated dataset, a stratified sample of approximately 200 claims was picked.

The CMS dataset has been selected as it best represents the structure and semantics of used life insurance claims without any loss of data privacy and reproducibility. Other popular data sets, including the IEEE-CIS fraud detection or Kaggle credit card fraud datasets, are financial transaction data, which exhibit very different behavior in features and time dynamics. Medical rich clinical datasets such as MIMIC-III or elcu do not have claim-level fraud labels and present identifiable data issues. CMS thus presents a suitable trade-off between accessibility, consistency of domains, and ethical standards that enables a significant cross-domain validation across the insurance and health care ecosystem.

In order to ensure a stable methodology, no adjustments were made to the preprocessing workflow (i.e., imputation, categorical encoding, normalisation, and SMOTE balancing). This was because no model-specific hyperparameter optimisation was done, and observed differences were only due to data distribution changes and not re-optimisation.

The results of comparative external validation are summarized in Table 15. Tab Transformer with SHAP retained the best level of discriminatory power (AUC = 0.689, F1 = 0.857), and was followed by Cat Boost with SHAP (AUC = 0.664, F1 = 0.841) and Bi-GRU with Attention (AUC = 0.647, F1 = 0.812). These values demonstrate a relatively small 2–3% decrease in AUC compared to the internal dataset, which attests to the stability of the unified interpretability framework to the heterogeneity of real-world claims (Table 18).

**Table 18:** External validation performance on the CMS medicare provider utilization dataset.

Model	Accuracy	Precision	Recall	F1-score	Avg.	Average
catBoost with SHAP	0.932	0.861	0.823	0.841	0.664	0.152
Bi-GRU with Attention	0.918	0.747	0.889	0.812	0.667	0.213
TabTransformer with SHAP	0.940	0.792	0.910	0.857	0.689	0.221

The prevalence ratio of fraud was further tested by adjusting it between 10% and 25%, and the number of unique provider identifiers was increased by 20. Tab Transformer with SHAP had the least difference in F1-score (± 2.4), followed by Bi-GRU with Attention (± 3.0) and Cat Boost with SHAP (± 3.5). The sensitivity analysis between the decision thresholds (0.4–0.6) gave a smooth monotonic precision-recall curve with no sudden drops in performance, which means that the models are stable even when there is a change in the underlying data distribution.

Collectively, these findings demonstrate that the suggested explainable cross-paradigm framework of fraud-detection is effective in generalizing to various datasets of claims, and is still as interpretable as it is in the original context. Scientific and ethical justifications support the use of the CMS dataset as a validation tool-its design depicts actual behavior under insurance claims, it is openly available to be reproducible, and its compatibility with regulations facilitates integrity in research. This third-party attestation forms an excellent basis for future large-scale proprietary life insurance data collections and federated information ecosystems.

### Significance testing for model robustness

To determine whether or not the performance differences between the models observed are statistically significant, two complementary tests were performed. To compare the mean performance differences, a paired t-test was used on the fold-wise accuracy and F1-scores of the five cross-validation splits. Moreover, the paired misclassification patterns of the model outputs of the same validation folds were compared with the help of the McNemar test. The two tests affirmed that CatBoost + SHAP and TabTransformer + SHAP have statistically significant enhancements (*p* < 0.05), but there is no statistically significant difference between the two models. These findings confirm that the performance trends in comparison that are found in the study do not occur by chance but are indicators of consistent behaviour of the models between folds.

## Discussion

### Influence of dataset characteristics on model

The model of detecting fraud is highly influenced by the dataset properties, such as class imbalance, class heterogeneity, and types of features. In this contribution, the dataset of 4000 life insurance claims, which has 83 features, reported an evident skewness between fraudulent and legitimate claims that affected the model behaviour. CatBoost with SHAP used the fact that it was able to handle categorical variables effectively and achieved high non-fraud accuracy (0.967), whereas Bi-GRU was more effective at detecting sequential patterns in the data, with the highest fraud recall rate (0.907). This important came with a higher rate of false positive demonstrating the modal sensitivity to imbalance class distribution. Similar results have been observed in previous studies. Boosting models had decreases in accuracy in the presence of imbalance unless the classes’ reweighting methods were used.

### Evidence in confusion matrices

The confusion matrix (Fig. 4) provides a more detailed view of the behavior of each model. CatBoost with SHAP maintained a low rate of false positives at the cost of a higher false negative rate, and therefore, it had a higher sensitivity. The Tab Transformer that used SHAP had the highest compromise, as it had both high recall and high precision with the highest fraud F1-score (0.872). This trend is in line with the previous results, emphasizing that the false negative is especially harmful in fraud detection since false negatives are associated with fraudulent payments that remain undetected, and false positives are associated with the extra verification expenses instead of the actual fraudulent payments that are detected^25,26^.

This is the correlation between Classification Reports and Model behaviour:

The classification report, as shown in Table 8, is consistent with the observation made based on the understanding of the confusion matrix. CatBoost SHAP offers the best in detecting valid claims with a non-fraud accuracy of 0.967. The Bi-GRU with attention that records a fraud recall of 0.907 fits well in case it is required to reduce the number of undetected fraud. In the meantime, the TabTransformer with SHAP had the most overall balance, a non-fraud accuracy of 0.984, and a fraud recall of 0.929, demonstrating how transformer models can be trained on complex feature interaction.

### Fraud-specific metric interpretation

The result shows that it is evident that metrics optimized to detect fraudulent activities present a more relevant evaluation of model performance on an unbalanced dataset compared to global accuracy. The results of the F-2 (2) scores indicate that the models based on sequential and contextual learning are more effective in capturing the minority-class behavior, which reflects the significance of the necessity to weigh the weight of recall more in such a situation when the cost of increased emphasis on recall, in case of fraud detection, is higher than the costs associated with further false positive investigations.

Similarly, the AUPRC values show the capacity of each model to preserve accuracy, with increasing recall showing its strength in prioritizing fraudulent claims over legitimate ones. Since these threshold metrics are not subject to a specific threshold, they provide a more realistic measure of operational screening performance, especially in those situations where a low false positive rate is essential^27,28^.

Besides numerical improvements in performance, the Bi-GRU with attention, and the SHAP-based features attributions indicated that the model has learnt a decision path, which corresponds to the known indicators of fraud, including high billing frequency and high claims cost. This evidence of interpretability justifies concluding that combining contextual modelling with a transparent explanation method enhances the reliability of an automated fraud detection method.

### Comparative model performance

The comparison of CatBoost and SHAP, Bi-GRU and attention, and TabTransformer and SHAP indicates that the models of traditional, sequence-based, and transformer-based models showed an apparent difference in performance. CatBoost with SHAP gave good baseline performance, especially on the legitimate claims, which is advantageous due to the ordered boosting algorithm that supports categorical variables. Its reduced recall of fraud cases, however, is an indication of a difficult ability to capture complex and nonlinear patterns in the data. Bi-GRU with attention enhanced the sensitivity in detecting fraud by exploiting its recurrent nature to learn the temporal relationships between claim histories. TabTransformer with SHAP was the most balanced model in terms of precision, recall, and F1-score, which can be explained by the ability of the transformer to capture higher-order relationships between categorical variables, using the attention mechanism.

### Feature importance and explainability

Fraud detection requires interpretability since stakeholders need to be assured of the logic behind automated decisions. The analysis using SHAP showed that claim amount, provider specialty, and diagnosis-procedure combinations were the most predictive factors. In the case of CatBoost with SHAP, a gain-based measure of feature importance was used, giving a simple list of the most important variables. Bi-GRU with Attention model is less interpretable; its attention weight visualization demonstrated the timely, unique segmentation of the claim sequence with the highest indication of fraudulent behaviour. TabTransformer with SHAP and attention weights provided to provide a dual-layer interpretability mechanism, not only revealing which features were important but also how they interrelated contextually within embedding spaces. This two-fold portrayal system took care of credibility in the eyes of regulators and decision-makers using such a system.

### Consistency of evaluation metrics

The distinction between AUC-based and accuracy metrics occurs naturally in imbalanced classification. Accuracy is the threshold-dependent correctness, dominated by the majority (non-fraud) class, whereas ROC-AUC and PR-AUC are measures of ranking performance with respect to threshold. In low-prevalence environments (fraud 18), models can have high accuracy by correctly classifying most of the data and have moderate ROC-AUC scores due to the contribution of minority-class true positives to the ranky curve. The PR-AUC values are lower since their baseline accuracy equals the fraud rate.

The cross-verification with the same validation folds ensured that under imbalanced data sets accuracy, ROC-AUC, and PR-AUC exhibit theoretically predicted relationships. Therefore, AUC-based metrics are numerically smaller; however, they all make sense when the effects of class imbalance and baseline precision are taken into account in the confusion-matrix statistics.

### Comparative evidence of other previous studies & benchmark with existing studies

**Table 19:** Comparison of health insurance fraud detection pattern models with previous research.

References	Methodology	Dataset type / size	Acc.	Recall	Interpretability	Key limitations
^18^	Ensemble (RF + Logistic Regression)	Insurance claim (~ 20k)	0.78	0.71	Moderate (feature gain)	Limited recall, bias toward the majority class
^21^	Deep Neural Network (DNN)	Medical claims (~ 15k)	0.81	0.82	Low	Black box, poor explainability
^24^	Graph Neural Networks (GNN)	Healthcare networks (~ 10k)	0.81	0.79	Limited (attention maps)	High computational cost
^19^	XGBoost with SMOTE balancing	Health insurance (~ 12k)	0.83	0.85	Moderate (feature importance)	Sensitive to synthetic oversampling
^20^	Autoencoder + Random Forest Hybrid	Medicare claims (~ 18k)	0.84	0.87	Low	Limited Interpretability
^22^	Transformer-based tabular fraud detection	Insurance claims (~ 25k)	0.86	0.89	High (attention weights)	Requires large-scale training
^23^	Current Study (TabTransformer + SHAP)	Life insurance (~ 4k, 83 features)	0.87	0.93	High (SHAP + attention)	The dataset size is smaller, and binary labels only

Benchmarking against prior work (Table 19) highlights the advantages of the proposed models. Ensemble methods have been reported with a recall of approximately 0.71 with logistic regression and random forest, and a deep neural network with a higher recall of 0.82, but interpreted less. The graphic neural networks that were used had high recall, but at a high computational cost. In autoencoders-RF hybrids, further enhanced recall, but with minimal interpretability on the feature level. In comparison, the TabTransformer with SHAP in this research had a recall of 0.929, a balance between interpretability, recall, and AUC. The recent TabTransformer using SHAP-based work was shown in an insurance claim, and it has good performance, but it has not used SHAP. The proposed TabTransformer + SHAP was applied to the task of detecting fraudulent health insurance. The model not only enhances the previous recall values but also offers the interpretive clarity that is needed in the application of the model in a regulated insurance fraud detection system.

### Interpretability and analysis of error

Applying the SHAP feature attribution and the pattern of attention weight revealed that the two patterns of explanations were unique but complementary. SHAP provides a global evaluation of the contribution of each feature to all samples, and attention focuses on short-term temporal effects, like recent claim activity or anomalies associated with a specific provider. When combined, they can give a more detailed picture of mode reasoning, but attention weights are an associative indicator and cannot be viewed as a causal explanation.

The similarity in the pattern of features between the SHAP and attention analyses revealed that both methods yielded significant cues related to fraud, including an amount variation of claims, policy duration, and high-frequency diagnostic codes. False positives (FP) were also generally associated with valid claims and large sums of money, and false negatives (FN) were more frequently associated with low-value claims or those that were not well documented. These observations indicate that further threshold tuning or calibration steps might be used to further enhance the model performance in an actual fraud detection workflow.

## Conclusion

The current paper introduces a single benchmarking model to model unbalanced and heterogeneous insurance-claim data that is able to be defined as a combination of CatBoost+SHAP, Bi-GRU+Attention, and TabTransformer+SHAP. TabTransformer gave the highest balanced outputs in 4000 sample datasets (18% fraud), with an accuracy of 0.951, a recall of 0.895, and an AUPRC of 0.938. To have valid cases, the accuracy (0.918) was higher with CatBoost+SHAP, but the sensitivity (F- 0.891) was higher with the Bi-GRU with attention. The two-layer interpretability paradigm, a combination of attention and weight insights and SHAP feature attribution, supported clear and auditable predictions based on logic. However, operational deployment should consider the latency, computational demand, and regulatory requirements since transformer architectures are more resource-consuming. Further testing is required to determine resiliency to distribution shift and temporal drift. In general, the results suggest that explainability combined with the variety of model architectures enhances the predictive reliability and working confidence, which is statistically supported by the Wilcoxon. (α < 0.05), which is a solid foundation to continue to develop larger, scalable systems of fraud detection.

## Future work

Various avenues of research that this unified benchmarking framework can take in the future are promising. To begin with, federated learning methods need to be explored so that collaboratively training models can be performed on distributed insurance data without risking sensitive policyholder information or regulatory regulations. Second, the framework needs to be scaled to real-time streaming infrastructure with platforms like Apache Kafka and Apache Flink to test model robustness to temporal distribution shift and concept drift in live claim pipelines. Third, by adding large language models and multimodal architectures to handle unstructured claim narratives and structured tabular features, fraud signals that are not seen by purely tabular models can be discovered. Fourth, inter-vertical transfer learning of domain-adaptive transfer of insurance would enhance the effectiveness of models and lessen the labelling load in new markets. Lastly, lightweight model distillation methods need to be considered to ensure transformer-based systems like TabTransformer can be practically deployed in resource-constrained production systems.

## Supplementary Information

Supplementary Information 1.

## Data Availability

The datasets used and/or analysed during the current study are available from the corresponding author on reasonable request.
